# Bioengineered Anthocyanin-Enriched Tomatoes: A Novel Approach to Colorectal Cancer Prevention

**DOI:** 10.3390/foods13182991

**Published:** 2024-09-21

**Authors:** Md Suzauddula, Kaori Kobayashi, Sunghun Park, Xiuzhi Susan Sun, Weiqun Wang

**Affiliations:** 1 Department of Food Nutrition Dietetics and Health, Kansas State University, Manhattan, KS 66506, USA; mdsuzauddula@gmail.com (M.S.); kobakaori@ksu.edu (K.K.); 2 Department of Horticulture and Nature Resources, Kansas State University, Manhattan, KS 66506, USA; shpark@ksu.edu; 3 Department of Grain Science and Industry, Kansas State University, Manhattan, KS 66506, USA; xss@ksu.edu

**Keywords:** anthocyanins, bioengineered tomatoes, anthocyanin-enriched tomatoes, colorectal cancer, cancer prevention

## Abstract

Colorectal cancer (CRC) remains a significant global health challenge, with barriers to effective prevention and treatment including tumor recurrence, chemoresistance, and limited overall survival rates. Anthocyanins, known for their strong anti-cancer properties, have shown promise in preventing and suppressing various cancers, including CRC. However, natural sources of anthocyanins often fail to provide sufficient quantities needed for therapeutic effects. Bioengineered crops, particularly anthocyanin-enriched tomatoes, offer a viable solution to enhance anthocyanin content. Given its large-scale production and consumption, tomatoes present an ideal target for bioengineering efforts aimed at increasing dietary anthocyanin intake. This review provides an overview of anthocyanins and their health benefits, elucidating the mechanisms by which anthocyanins modulate the transcription factors involved in CRC development. It also examines case studies demonstrating the successful bioengineering of tomatoes to boost anthocyanin levels. Furthermore, the review discusses the effects of anthocyanin extracts from bioengineered tomatoes on CRC prevention, highlighting their role in altering metabolic pathways and reducing tumor-related inflammation. Finally, this review addresses the challenges associated with bioengineering tomatoes and proposes future research directions to optimize anthocyanin enrichment in tomatoes.

## 1. Introduction

CRC has now become a world health issue; it is the third most common malignancy and the second most leading reason for cancer-related deaths [1]. The global burden of CRC has increased substantially, with a 157% rise in incidence from 1990 to 2019, resulting in 2.17 million cases in 2019 [2]. Such elevation is particularly evident in developing regions, such as East Asia and Middle Socio-Demographic Index Regions, where the burden is growing the fastest [3]. In China, CRC poses a severe challenge, with 560,000 new cases and 290,000 deaths in 2020 alone, and projections indicating a 64% increase in incident cases by 2040 [1]. A summary report on CRC has been published by the Surveillance, Epidemiology, and End Results program under the National Cancer Institute. The incidence of CRC in the US was reported at a rate of 36.5 cases per 100,000 individuals annually. Although the rate of people being diagnosed with colon or rectal cancer each year has dropped overall since the mid-1980s in the US, it is still a threatening and raging disease in the world as a whole. All types of cancer have some similar patterns and nature of growth and spread. When a cancer is identified solely within the original site of occurrence, it is categorized as localized (sometimes denoted as stage 1). Conversely, if it has disseminated to another area of the body, it is classified as either regional or distant. In the case of colorectal cancer, the distribution among local stage diagnoses stands at 34.8%, the regional stage at 36%, and the distant stage at 23%. Five years of relative survival rates for localized, regional, and distant CRC were reported as 91.1%, 73.7%, and 15.7%, respectively. The interplay between these genetic predispositions and environmental exposures underscores the multifactorial nature of CRC etiology. Effective screening programs and early diagnosis, along with lifestyle modifications, are essential to mitigate the rising incidence and improve survival rates [4,5].

Cancer treatment has a variety of technologies and methodologies, including surgery, chemotherapy, radiation therapy, and emerging biotechnologies like immunotherapy and targeted therapy [6]. While these treatments and strategies are crucial for managing and potentially curing cancer, they often come with significant side effects and complications [7,8]. On average, 10% of colorectal surgery cases lead to small bowel obstruction (SBO) due to peritoneal adhesions post-surgery. The recurrence of adhesive SBO is linked to a lower survival rate [9]. The late adverse effects of radiotherapy on CRC include bowel obstructions and dysfunction, manifesting through symptoms such as fecal incontinence, lose or solid stools, gas, evacuation difficulties, and sexual dysfunction [10]. In contrast, plant-based compounds exhibit several special outcomes of CRC treatment. For example, plant-based compounds effectively manage chemotherapy-related side effects, delay tumor growth, and modulate multiple molecular pathways linked with multidrug resistance [11,12]. Within plants, one can find bioactive phytochemicals including flavonoids, polyphenols, and catechins, which play a crucial character in reducing tumor cell propagation by obstructing cell cycle checkpoints and fostering apoptosis [11].

Anthocyanins represent water-soluble pigments within the phenolic compound category. Berries, currants, grapes, and specific tropical fruits exhibit a notable abundance of anthocyanins. The presence of high anthocyanin content is characteristic of leafy vegetables, grains, roots, and tubers displaying hues ranging from red to purplish-blue [13]. The potential anti-cancer effects of anthocyanin may emanate from a variety of biological processes, encompassing anti-oxidative stress, anti-inflammatory actions, anti-mutagenesis properties, the stimulation of differentiation, the suppression of proliferation, the arrest of the cell cycle, and the induction of apoptosis, alongside anti-invasion, anti-metastasis, anti-angiogenesis effects, and the sensitization of cancer cells to chemotherapy [14]. Several studies proved the power of anthocyanin to fight against CRC [15,16]. In particular, the consumption of anthocyanin extracts from strawberries, black raspberries, tart cherries, and blackberries with higher doses could significantly decrease tumor proliferation [17]. The higher doses are equal to a higher number of fruits, which may not be affordable for middle- and low-income families, especially from those countries where such fruits need to be imported. While berries are renowned for their high anthocyanin content, several other fruits and vegetables are often less expensive and contain anthocyanin, but in trace amounts [18]. The biosynthesis of anthocyanins among those plants can be possibly manipulated by bioengineering. For instance, the overexpression of *MYB* genes like *VvmybA1* from red grape and *Ruby* from ‘Moro’ blood orange in Mexican lime has resulted in significant anthocyanin accumulation in leaves, flowers, and fruits, thereby increasing the antioxidant activity of the transgenic plants [19,20]. Additionally, the development of anthocyanin-rich cultivars such as the ‘Sun Black’ tomato in Europe and the ‘Kufri Neelkanth’ purple potato in India demonstrates the successful application of genetic engineering in enhancing anthocyanin content without compromising yield [19].

To increase the affordable supply of anthocyanin to all classes of people around the globe, a frequently consumed, less expensive, and easily cultivatable crop could be subjected to bioengineering. Consequently, the target population could have access and purchase capacity. Tomato (*Solanum lycopersicum*), second only to potatoes in terms of vegetable consumption in the US, presents a promising opportunity for the consideration of bioengineering to enhance anthocyanin due to its status as one of the most extensively studied crop species at the metabolomic level. Thus, it establishes itself as a primary model for the examination of flavonoids and phenylpropanoids [21,22]. Bioengineered tomato containing high anthocyanin levels was documented in 2008, and US government agencies recently approved bioengineered purple tomatoes for market, a significant advancement for enriching human diets. On top of that, EU legislation started approving bioengineered crops in various sectors, with slow and challenging approval processes [21]. To understand the importance of bioengineered anthocyanin-enriched tomatoes to face CRC, this review focused on accumulating available knowledge and information on the role of bioengineered anthocyanin-enriched tomatoes in preventing CRC and its inflammation. In addition, this review is committed to showcasing the mechanism to prevent CRC through anthocyanin extracts from bioengineered tomatoes as well as other natural sources.

## 2. Anthocyanins

### 2.1. Natural Sources of Anthocyanin

**Figure 1:**
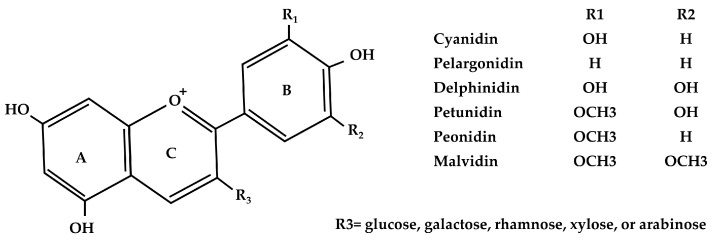
Molecular structure of common anthocyanins and anthocyanidins.

Anthocyanins have been historically documented as “colored cell sap” for many centuries. In 1835, a German botanist officially designated this compound [23]. Within the classification of secondary phytochemicals termed flavonoids, anthocyanin is identified as a specific subset [24]. Anthocyanin produces a diverse range of colors including purple, pink, blue, and red in plant organisms, namely flowers, vegetables, and fruits [24]. The modification of anthocyanin’s chemical structure through processes like hydroxylation, methylation, glycosylation, and acylation enhances their color variations and stability. The structure of the major type of anthocyanin is presented in Figure 1. The color of anthocyanins transitions to blue as the number of hydroxyl groups in the B-ring increases, whereas methylation induces a shift toward the red end of the spectrum. The methylation of the B-ring is important for reducing oxidation susceptibility and enhancing anthocyanin stability [24]. Anthocyanins have a unique UV−Vis absorption pattern for their extended chromophore with eight double bonds alone with a positive charge on the O_2_ ring in an acidic environment. This pattern shows an absorption peak among the visible range of 465 nm and 550 nm. It also gives a peak at another absorption between 270 nm and 280 nm [25]. Naturally, a rich diversity of over 635 distinct anthocyanins has been identified [26]. Among these, six primary anthocyanidins—cyanidin, pelargonidin, delphinidin, petunidin, peonidin, and malvidin—dominate, collectively representing about 90% of all identified anthocyanins [27]. The distribution of these anthocyanidins in natural sources is typically 30% cyanidin, 22% delphinidin, 18% pelargonidin, 7.5% peonidin, 7.5% malvidin, and 5% petunidin [28]. A list of plant sources that contain comparatively higher amounts of anthocyanin is presented in Table 1. Although anthocyanins can be isolated from various plants like sweet potato, hibiscus, corn, cabbage, and carrot, berries are primarily recognized as the main reservoir of anthocyanins and provide about 29.17% of anthocyanin of all sources. Following berries, other significant sources of anthocyanins include grape, sour cherry, black rice, and black carrot. Furthermore, purple maize, pomegranate fruit, saffron, banana, and mao fruit also serve as notable sources of anthocyanins [29].

### 2.2. Factors That Affect the Yield of Anthocyanin

The yield of anthocyanin, a pigment with significant antioxidant properties, is influenced by several factors, such as the type of solvent used, soil temperature, plant preparation methods, extraction techniques, cofactor selection, and environmental conditions [50].

#### 2.2.1. Extraction Techniques

Likewise, extraction techniques influence anthocyanin yields because different extraction techniques have different principles which alter the extraction yield and quality [51]. For instance, in the study on freeze-dried strawberry puree, two chloroform-based methods yielded the highest anthocyanin content, while an extraction solvent MeOH:H_2_O: HCl extracted an intermediate amount of anthocyanin, and the pH differential method yielded the lowest anthocyanin content, despite its shorter processing time [52]. In a separate investigation, the utilization of enzyme–microwave-assisted extraction on *Hibiscus sabdariffa* L. led to a recorded anthocyanin output of 15.37 ± 0.41 mg/g, whereas a microwave-assisted extraction yielded 13.01 ± 0.22 mg/g, ultrasound-assisted extraction yielded 11.73 ± 0.28 mg/g, and enzyme-assisted extraction led to an output of 11.06 ± 0.33 mg/g, correspondingly [53]. Similarly, ultrasound-assisted extraction of *Myrciaria cauliflora* Berg.’s skin showed that increasing the extraction time improved anthocyanin yield from 200 mg (at 1st min) to 400 mg (at 3rd min) per 100 g DW and antioxidant capacity, making it the most effective method compared to conventional and high-pressure extractions [54]. Optimized ultrasound-assisted extraction demonstrated a yield of 311 ± 5 mg of anthocyanin from 100 g of *Hibiscus sabdariffa* L. calyces, surpassing the yield obtained through the traditional extraction method [55]. On top of that, another study on *Hibiscus rosa-sinensis* petals revealed that ultrasound-assisted extraction yielded 179.32 mg/L of anthocyanins, outperforming microwave-assisted extraction at 155.45 mg/L and conventional extraction at 100.88 mg/L [56]. A highly efficient separation methodology has been developed, referred to as counter-current chromatography (CCC). This technique demonstrated extraction purities exceeding 90% for specific anthocyanins, including delphinidin-3-glucoside, cyanidin-3-rutinoside, and delphinidin-3-rutinoside [57]. In another investigation, anthocyanins were extracted from strawberries utilizing high-speed counter-current chromatography, employing a biphasic solvent system composed of tert-butyl methyl ether, n-butanol, acetonitrile, water, and trifluoroacetic acid in a ratio of 2.5:2.0:2.5:5.0:1.0%. The identified anthocyanins included pelargonidin-3-rutinoside, cyanidin-3-glucoside, and pelargonidin-3-glucoside, exhibiting purities of 95.6%, 96.2%, and 99.3%, respectively [58]. These findings collectively highlight that advanced extraction techniques, particularly those involving ultrasound, enzyme–microwave assistance, and current, tend to enhance anthocyanin yields significantly compared to conventional methods, with specific numeric data underscoring the superior efficiency of these modern techniques.

#### 2.2.2. Extraction Solvents

The extraction of anthocyanins found in various plants is significantly influenced by the choice of extraction solvents. It affects both yield and stability. Among the various solvents that were investigated, chloroform–methanol, ethanol, and methanol showed the most significant degree of anthocyanin extraction capacity, approximately 10–12 A/g FW. Solvents like methanol–water, pH differential buffers, and acetone demonstrated a moderate efficiency in extracting anthocyanins, ranging from 6.8 to 8.8 A/g FW. Moreover, a combination of different solvents with water displayed the least effective solvents, with an extraction yield of less than 5 A/g FW of strawberry puree [50]. In contrast, only distilled water demonstrated superior solvency compared to 96% ethanol for the extraction of anthocyanins from Butterfly Pea flowers. The water extraction method, in particular, resulted in the production of anthocyanin, 4841 mg/g Butterfly Pea [59]. Novel techniques such as microwave–ultrasonic-assisted extraction (MUAE) in combination with natural deep eutectic solvents (DESs) have displayed potential in the efficient retrieval of anthocyanins from purple perilla leaves, attaining a maximum output of 619.62 mg/100 g under ideal conditions [60]. Likewise, the utilization of natural deep eutectic solvents (NaDES), specifically formulations involving choline chloride and malic acid, in conjunction with ultrasonication-assisted extraction, has proven to be successful in the retrieval of chokeberry anthocyanins. Moreover, additional enhancements have been noted through the integration of hydroxypropyl-β-cyclodextrin (HP-β-CD) to boost both yield and stability. The extraction efficiency experienced enhancement with HP-β-CD concentrations of up to 3% (*w*/*w*) [61]. Additionally, DESs based on choline chloride and xylitol significantly increased the anthocyanin yield from *Euterpe edulis Mart.* The increase in fruit pulp by 42% compared to methanolic extraction also demonstrated superior antioxidant capacity and slower degradation under heat and light [62].

#### 2.2.3. Extraction pH

The extraction pH showed influences on both the quantity and quality of anthocyanins pulled out from various plant sources. For instance, in the study on extracted anthocyanin from *Hibiscus rosa-sinensis*, scientists compared the outcome of pH 2 and pH 4, revealing that the highest total anthocyanin (cyanidin-3-glucoside) yields were 8.33 mg/L at pH 2 and 9.56 mg/L at pH 4 [63]. Similarly, in the extraction of anthocyanins from water caltrop hull, the optimal pH was found to be 4.49, which resulted in a 70.3% anthocyanin extraction efficiency [64]. In red radish, a lower pH of 2.5 yielded higher anthocyanin content (98.02 mg/100 g, FW) compared to pH 4.5, which also resulted in a lighter color and higher polymeric color percentage, suggesting that lower pH conditions are more favorable for maintaining anthocyanin stability and color intensity [65]. The type of anthocyanin extracted also varies with pH; for example, in strawberries, pelargonidin was quantified as a major abundance of anthocyanin in methanol–chloroform solvent, while delphinidin dominated in other category solvents, indicating that solvent pH can alter the anthocyanin profile [50]. Additionally, the pH-sensitive film study demonstrated that anthocyanin extracts from *Lycium ruthenicum* Murr. changed color across a pH range of 2.0 to 10.0, further illustrating the impact of pH on anthocyanin stability and visual properties [66]. Overall, these studies collectively highlight that lower pH conditions generally enhance anthocyanin yield and stability, while higher pH conditions can lead to different anthocyanin profiles and potentially higher extraction efficiencies, depending on the plant source and extraction method used.

#### 2.2.4. Cultivar and Genetic Factors

Genetic diversity and bioengineering have significantly impacted anthocyanin yield in various fruits and vegetables, as evidenced by multiple studies. In blueberries and bilberries, the upregulation of the transcriptional activator MYBA1 in conjunction with *VmANS* resulted in a threefold elevation in anthocyanin concentration, underscoring the involvement of specific structural genes in anthocyanin biosynthesis [67]. Similarly, in the context of olive fruits, specifically the Carolea and Tondina varieties, research findings indicated that the overall concentration of anthocyanins was greater in the ‘Tondina’ cultivar compared to the ‘Carolea’ cultivar. Inconsistencies were also observed concerning the genetic composition [68]. In the case of tomatoes, the ectopic expression of the MYB-like transcription factor *PhAN4* triggered the synthesis of atypical anthocyanins like delphinidin and petunidin derivatives, while augmenting the plant’s capacity to combat oxidative stress, a crucial aspect for applications in modern agriculture [69]. Examination of the gene expression patterns associated with anthocyanin production in violet tomatoes indicated that key regulatory genes like *SlANT1* and *SlAN1* are pivotal in activating structural genes, thereby facilitating anthocyanin deposition in the fruit skin [70]. In woodland strawberries, altering the expression of the R2R3 *MYB* activator *MYB10* significantly affected anthocyanin concentrations, with overexpression increasing and knockdown decreasing the levels of cyanidin and pelargonidin glucosides [71]. The incorporation of the *SmMYB1* gene into an anthocyanin-deficient eggplant variety resulted in elevated levels of anthocyanins in multiple plant parts, notably the fruit pulp, alongside enhanced resistance to frost stress [72]. In horticulture crops, the synchronized expression of the structural genes implicated in anthocyanin synthesis is impacted by external elements like light and temperature, which have an impact on the expression of regulatory genes [73]. The enhancement of regulatory and structural genes in blueberries during the later phases of fruit growth resulted in a notable accumulation of anthocyanin, causing the fruit to exhibit a blue color [74]. Within Asian pears, the transcription factor *PyMYB10* belonging to the R2R3-MYB family was recognized as a key player in the regulation of anthocyanin production, displaying a positive association between its expression and the anthocyanin levels in red-skinned varieties [75]. These studies collectively demonstrate that the targeted manipulation of gene expression and bioengineering can effectively enhance anthocyanin yield in fruits and vegetables, providing valuable insights for breeding programs aimed at improving nutritional properties and stress tolerance.

### 2.3. Health Benefits and Functional Properties of Anthocyanins

Anthocyanin consumption offers a range of specific health benefits, particularly in controlling and preventing chronic diseases.

#### 2.3.1. Circulatory-Related Health Issues

Clinical trials have shown that anthocyanin-rich foods significantly reduce fasting blood glucose by a notable percentage, HbA1c by 2%, total cholesterol by 1%, triglycerides by 0.3%, and low-density lipoprotein by 0.5%, while increasing high-density lipoprotein by 0.3% [76]. Anthocyanins from roselle administered to Wistar rats resulted in a decreased heart rate and blood pressure across varying doses of 50, 100, and 200 mg/kg body weight. The findings indicate that the impact of anthocyanins on rats implies a notable antihypertensive efficacy, potentially through the modulation of elements within the renin–angiotensin–aldosterone system [77]. Anthocyanin treatment at 10 mg/kg, 0.4 μL/h decreased a salt-induced rise in heart rate and blood pressure. Additionally, it hindered sympathetic nerve activation by lowering LF/HF and raising RMSSD and SDNN levels in hypertension induced by high salt [78]. Clinical trials have shown that anthocyanin supplementation significantly reduces body mass index by an average of 0.36 kg/m^2^, and is particularly effective at doses of 300 mg/day or less over four weeks [79]. Moreover, anthocyanins have demonstrated the ability to lower fasting blood glucose, HbA1c, total cholesterol, triglycerides, and levels of low-density lipoprotein, while simultaneously elevating high-density lipoprotein levels in various clinical and pre-clinical investigations. These findings suggest a beneficial influence on metabolic health indicators linked to obesity [76].

#### 2.3.2. Chronic Liver Disease

Research conducted on 2288 adult participants from the United States revealed that a higher consumption of anthocyanins was found to be significantly linked to a reduced likelihood of Non-Alcoholic Fatty Liver Disease. In an investigation, hepatic fibrosis was induced in a mouse model by CCL4, where anthocyanin treatment with doses of 100 and 200 mg/kg notably reduced hepatic fibrosis, inhibited hepatic stellate cell proliferation, and reversed blocked autophagic flux through the modulation of the circ_0000623/miR-351-5p/TFEB pathway [76]. Black raspberry anthocyanins also showed protective effects against adrenoleukodystrophy by ameliorating serum biochemical parameters, reducing liver damage, and promoting apoptosis in HepG2 cells through the TGF-β and NF-κB pathways [80]. Black raspberry anthocyanins (25, 50, 100, 150, and 200 μg/mL) and cyanidin-3-O-rutinoside (10, 20, 40, 80, and 100 μM) demonstrated cytotoxic effects on t-HSC/Cl-6, HepG2, and Hep3B cells, as well as induced cell death programming in HepG2 cells. These compounds were found to downregulate Bcl-2 protein expression, upregulate Bax levels, and facilitate cytochrome C release, cleaved caspase-3, cleaved caspase-9, and cleaved PARP in HepG2 cells [80]. The findings of the study indicate that the preventive impact of anthocyanin from black raspberry on alcoholic liver disease is mediated through antioxidative and apoptotic pathways.

#### 2.3.3. Immunity Responses

In animal research, anthocyanin-enriched diets have demonstrated the ability to restore cellular immunity parameters, specifically the proportion of T helper cells (CD3+CD4+) and T cytotoxic lymphocytes (CD3+CD8+), which are commonly disturbed in cases of obesity [81]. BALB/c mice with leukemia were provided with a diet containing anthocyanins extracted from purple glutinous indica rice at doses of 0, 20, 50, or 100 mg/kg over three weeks. The supplementation of anthocyanins was observed to stimulate the populations of CD3 (T cell), CD19 (B cell), CD11b (monocyte), and Mac-3 (macrophage) in the leukemia-afflicted mice. Moreover, treatment with AUPGA was found to enhance macrophage phagocytosis while reducing the activity of NK cells [82]. Fluorosis was induced in four-week-old healthy male Wistar rats through the administration of fluoride for 90 days. The experimental intervention involved the administration of anthocyanin extracted from blueberries at a concentration of 100 mg/kg body weight per day. The levels of IgG and IL-1 markers exhibited a significant increase in the groups treated with anthocyanins. These immunoglobulins and cytokines play a crucial role in both the immune response and immune regulation [83]. Therefore, the results indicate the potential of anthocyanins in enhancing immune responses in living organisms.

#### 2.3.4. Cancers

Anthocyanins’ broad antitumor activities include anti-inflammatory, antioxidant, anti-metastasis, and anti-mutagenesis effects and also reverse drug resistance and increasing sensitivity to chemotherapy, making them versatile agents in cancer treatment [84]. The MCF 7 breast cancer cell line was treated with anthocyanins (50 µg/mL) from *Cordyline australis* (cabbage tree). The study revealed that anthocyanin treatment with 65 ± 2.1% cytotoxicity induced profound activities in caspase-3 (157%), caspase-8 (142%), and caspase-9 (147%). These findings indicated anthocyanin’s potential in promoting apoptosis and inhibiting cancer cell proliferation [85]. Moreover, a human study with 25 CRC patients who received oral bilberry extract (containing 0.5–2.0 g of anthocyanins) for 7 days demonstrated a dose-dependent existence of anthocyanins in circulation and tumor tissues. The concentrations reached around 179 ng/g of tumor tissue at the highest dose. This treatment resulted in a 7% reduction in tumor cell proliferation [15]. Furthermore, dietary factors rich in anthocyanins, such as berries and grapes, have been associated with protective effects against CRC due to their high levels of cancer-fighting phytochemicals [86]. In another animal study, phycocyanin, a pigment–protein complex, demonstrated anti-CRC activities by reducing tumor numbers and inhibiting epithelial cell proliferation, alongside altering the composition of the microbiota inside the gut and affecting the IL-17 signaling pathway [87]. Collectively, these findings underscore the multifaceted role of anthocyanins in cancer prevention, highlighting their potential as complementary therapeutic agents in oncology. Future investigation is necessary to elucidate the precise mechanisms (molecular and cellular levels), optimal dosages, and available sources for their effective use in cancer prevention and treatment.

### 2.4. Bioaccessibility and Absorption of Anthocyanin

Based on the above discussion, without any doubt, anthocyanin has numerous health benefits and functional properties. Furthermore, to maximize the benefits of anthocyanin to protect our health, the bioaccessibility and bioavailability through the gastrointestinal tract (GIT) of this compound should be kept in mind [88]. This is because the plant-based matrix, for instance, the cell wall and fibrous tissue structure, could affect the absorption of starch, lipids, proteins, and functional phytochemicals [89]. An in vitro gastrointestinal model was used and demonstrated anthocyanin’s bioaccessibility from purple carrots and potatoes sunk at 44.62 and 71.8%, respectively [90]. On top of that, the total phenolic and carotenoid contents and the antioxidant activities of purple tomato were significantly reduced by 37–72%, and degradation seemed to have occurred during the in vitro digestion [91]. Although the evidence showed lower bioaccessibility, anthocyanin could rapidly be absorbed from the stomach and enter the systemic circulation within minutes. Then, they reach maximum concentrations after a few hours but rapidly decline afterwards [92]. Anthocyanins are extensively metabolized before entering systemic circulation since some of their metabolites are generally more concentrated than the original compounds [93,94]. Effective strategies that could enhance the bioavailability of anthocyanin through the oral consumption of raw plants need to be discovered. At this point, increasing the oral consumption of anthocyanin-enriched plant organs could be an easy approach.

On the other hand, anthocyanin could be extracted from anthocyanin-enriched plant organs which could minimize the limitation identified due to the plant-based matrix. However, anthocyanins exhibit sensitivity to pH variations and may degrade substantially within the digestive system, especially during the acidic gastric phase. Such degradation influences their bioavailability to the tissue and the organ’s biological activity [95]. An in vitro study was conducted on the availability of anthocyanin extracted from raspberry in GIT and revealed that only around 5% anthocyanin could be available in serum available material [96]. However, delivering a significant and effective dose of anthocyanin could maximize its potential, where modern methods such as encapsulating could be adopted. Recent studies demonstrated that microencapsulated anthocyanin not only exhibited a superior antioxidant capacity but also enhanced anthocyanin retention compared to nonencapsulated anthocyanins and cyanidin-3-glucoside in both the gastric and intestinal phases [97]. On top of that, a study on microencapsulated (by whey protein/fructo-oligosaccharide) anthocyanins (extracted from black soybean peels) found a higher concentration in circulation. This reflects the enhancement of absorption of anthocyanins by microencapsulation, which might be the presence of encapsulated materials that made more anthocyanin transportation to the absorption system [98]. Overall, ensuring the bioavailability of anthocyanin absorption by GIT and adapting appropriate anthocyanin delivery methods like encapsulation could enhance the health benefits and functional properties of anthocyanins.

## 3. Effect of Anthocyanin on Modulation of the Signaling Pathways in CRC

CRC is a multifaceted disease influenced by the alteration of several key signaling pathways. Each pathway contributes uniquely as well as collaboratively to the initiation, expansion, and progression of CRC. The NFκB signaling pathway is one of them, and it is significantly involved in CRC through its role in inflammation. The overactivation of this pathway triggers the upregulation of genes such as *IL1B*, *CXCL8*, *IL1A*, and *CSF2*, which are well known for tumor growth and poor differentiation [99]. Similarly, the Wnt/β-catenin signaling pathway is also found to be hyperactivated in almost all CRC patients. This pathway influences the propagation of cancer stem cells, chemoresistance, epithelial–mesenchymal transition, and metastasis [100,101]. On top of that, this pathway’s interaction with the PI3K/AKT pathway further enhances CRC progression. Such conditions promote cellular migration and proliferation, and resistance to PI3K or AKT inhibitors can be mitigated by targeting Wnt/β-catenin signaling [102,103]. Another signaling pathway known as the JAK/STAT pathway, along with other pathways, is found to be dysregulated in CRC stem cells, contributing to their survival, proliferation, and self-renewal properties, which are critical for disease progression and reappearance [104]. Moreover, the p53 signaling pathway is also often mutated in CRC, plays a key part in cell cycle regulation and apoptosis, and its inactivation leads to unchecked cellular proliferation and tumor growth. Another pathway is known as the PI3K/AKT/mTOR pathway, which is also a critical axis in CRC, by controlling cellular autophagy, metabolism, metastasis, and cell cycle progression. Its activation is related to poor prognosis, and the inhibitors targeting this pathway are being explored, although resistance mechanisms often emerge [105]. The TGFβ/BMPs signaling pathway is involved in CRC through its dual role in tumor suppression and promotion, depending on the context and stage of cancer. The dysregulation of this pathway can lead to enhanced epithelial–mesenchymal transition and metastasis. Collectively, these pathways form a complex network that drives CRC pathogenesis, and understanding their interplay is essential for establishing noble and effective therapeutic strategies. For instance, targeting the Wnt/β-catenin pathway in combination with PI3K/AKT inhibitors has shown the potential to overwhelm drug resistance and improve treatment results [103,106]. Additionally, controlling the NFκB and JAK/STAT pathways could provide new avenues for targeting CRC stem cells and reducing tumor recurrence [99,104]. Therefore, a comprehensive approach that considers the intricate interactions among these pathways is crucial for advancing CRC therapy and improving patient prognosis. Treatment with anthocyanin showed promising protective effects against CRC through altering the above signaling pathways. The effect of anthocyanin on the modulation of the transcription factors that are involved in forming CRC has been presented in Table 2.

### 3.1. Anthocyanin on NFκB Signaling Pathway

**Figure 2:**
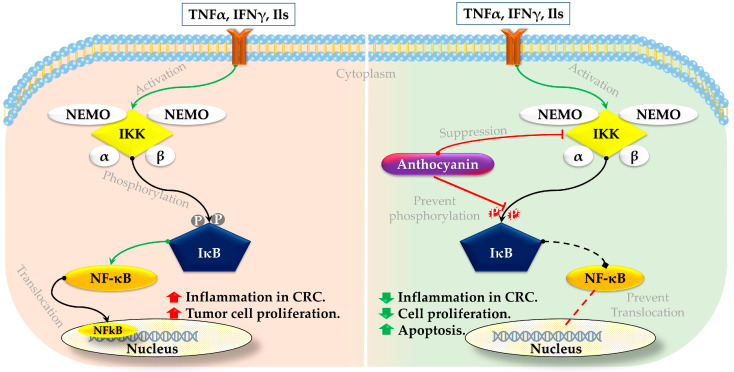
Effect of anthocyanin on NFκB signaling pathway in CRC. Adopted and modified from [125,126,128].

Studies have found that anthocyanin treatment has promising effects on the NFκB signaling pathway in CRC [86]. The beneficial mechanism involves the suppression of IκB kinase (IKK) activity, preventing phosphorylation and the subsequent degradation of IκB proteins [125,126]. Such inhibition results in holding the NFκB in the cytoplasm, which prevents NFκB’s translocation to the nucleus where it would otherwise activate the transcription of genes involved in cancer inflammation, cell proliferation, and survival [127] (Figure 2). By inhibiting this pathway, anthocyanins help reduce inflammation and tumor growth in CRC cells.

In an investigation, HCT116, HT29, and SW620 cells (human colon cancer cell line) were treated with 10, 25, 50, and 100 µM of anthocyanin (Cyanidin chloride) for 24, 48, and 72 h. The results revealed that treatment with anthocyanin induced apoptosis and significantly impeded cellular proliferation and colony formation in those three colon cancer cells. In particular, anthocyanin suppressed the NFκB signaling pathway while enhancing the activation of the nuclear factor erythroid 2-related factor 2 (Nrf2) pathway in colon cancer cells [126]. Another study observed that HCT116 to anthocyanidin (delphinidin) at a concentration of 30–240 µM for 48 h resulted in decreased cell viability, the initiation of apoptosis, PARP cleavage, the activation of caspases-3, -8, and -9, the upregulation of Bax with a simultaneous downregulation of the Bcl-2 protein, and cell cycle arrest at the G2/M phase. Introducing anthocyanin to HCT116 cells also led to the inhibition of IKKα, phosphorylation and degradation of IκBα, phosphorylation of NFκB/p65 at Ser536, translocation of NFκB/p65 to the nucleus, NFκB/p65 binding to DNA, and induction of NFκB’s transcription. The study concludes that the use of anthocyanin on HCT116 cells can block the NFκB pathway, resulting in apoptosis and arrest at the G2/M phase in CRC [129].

### 3.2. Anthocyanin on Wnt/β-Catenin Signaling Pathway

**Figure 3:**
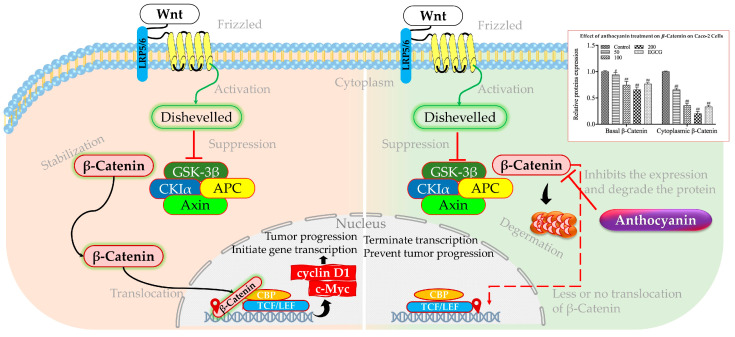
Schematic representation of activated and inhibited Wnt/β-catenin pathway. Hashes indicate significant differences with control group only at the (#) *p* ≤ 0.05 and (##) *p* ≤ 0.01 levels, respectively. Adopted and modified from [101,136,139].

The crosstalk between inflammatory signaling and the Wnt/β-catenin pathway leads to the relocation of β-catenin to the cell nucleus, subsequently stimulating the synthesis of cancer-promoting proteins such as cyclin D1 and c-Myc (Figure 3). Moreover, in many human cancers such as CRC, up to 70–80% elevation of c-Myc expression was observed [130]. This process leads to the proliferation of stem cells while impeding their differentiation [131]. The overactivation of Wnt signaling is a common feature in nearly all cases of CRC and significantly contributes to various cancer-related activities such as the spread of cancer stem cells, angiogenesis, epithelial–mesenchymal transition, resistance to chemotherapy, and metastasis [101]. Other transcription factors also could influence the Wnt/β-catenin signaling pathway. A case in point is FOXO3a, which typically functions as a suppressor of tumor growth and has been shown to impede the Wnt/β-catenin pathway in various cancer forms. On top of that, in cervical carcinoma, FOXO3a overexpression inhibits cell invasion and migration by negatively regulating the Wnt/β-catenin pathway, suggesting a comparable mechanism could be at play in CRC [132]. The interaction between FOXO3a and microRNAs also influences the Wnt/β-catenin pathway, as seen in various cancers where microRNAs either synergize with or antagonize FOXO3a to modulate tumor growth and metastasis [133]. Another transcription factor called RUNX3 downregulates c-Myc expression, a key oncogene, through two parallel pathways: directly at the transcriptional level and by attenuating β-catenin/TCFs, downstream of BMPs in CRC cells [134]. RUNX3 also binds to the METTL3 promoter, activating circMETTL3 transcription, which in turn acts as an miR-107 sponge to regulate PER3 signaling, thereby restraining CRC development and metastasis [135].

Anthocyanins have been found to interact with the Wnt/β-catenin signaling pathway, a critical regulator of cell growth and differentiation that is frequently disrupted in colorectal cancer [136]. The mechanism underlying this interaction involves the inhibition of the pathway by anthocyanins, which reduces the levels of β-catenin, thus preventing its cytoplasmic accumulation and subsequent movement into the nucleus (Figure 3) [137]. A study utilizing anthocyanin-enriched purple-fleshed potatoes was conducted to investigate its impact on colon cancer. It was discovered that the anthocyanin extract derived from purple potatoes exhibited a greater suppression of cytoplasmic and nuclear β-catenin levels compared to sulindac in colon cancer stem cells with active p53 and p53 that were attenuated by shRNA. Furthermore, the anthocyanin extract also effectively inhibited downstream targets of the Wnt/β-catenin pathway, namely c-Myc and cyclin D1, in colon cancer stem cells with functional p53 and p53 that were attenuated by shRNA. These findings provide evidence supporting the inhibition of β-catenin nuclear translocation by anthocyanin extract, thereby restraining the growth of colon cancer stem cells [138].

HCT116 cells were subjected to treatment with hawthorn proanthocyanidin extract of 200, 250, and 300 μg/mL. The findings indicated a reduction in the mRNA expression of Wnt1, c-Myc, and Slug in the treatment cohort, which was opposite to the control group. Through Western blot analysis, there was a significant decrease in the quantities of c-Myc and Snail protein of HCT116 cells that were exposed to high hawthorn proanthocyanidin extract for 48 h [140]. Moreover, a group of adult male Sprague Dawley rats was subjected to 4 mg N-methylnitrosourea per ml water/mouse to induce CRC, followed by intraperitoneal administration of either 12.5 mg/kg 5-fluorouracil or oral gavage 2.25 to 4.5 g/kg of Punica peel extract. N-MNU increased the mRNA expression of the β-Catenin, K-ras, and C-Myc genes in colon tissues, as compared to the negative control group. Treatment with Punica peel extract effectively decreased the expression levels of the investigated genes (*β-Catenin*, *K-ras*, and *C-Myc*) in a dose-dependent manner compared to the *N*-MNU group (untreated). The potential mechanism of action for these effects of the Punica peel extract, which include apoptosis promotion, inflammation mitigation, and suppression of tumor cell proliferation in vivo, may involve the inhibition of the Wnt/β-Catenin signaling pathway [141].

### 3.3. Anthocyanin on JAK/STAT Signaling Pathway

The relationship between CRC and the JAK/STAT signaling pathway is multifaceted and significant in understanding the disease’s progression and treatment resistance. The JAK/STAT pathway maintains a crucial transmembrane signal transduction mechanism activated by various cytokines, growth factors, and other molecules, driving processes such as proliferation, immune response, and malignancy [142]. The dysregulation of this pathway is strongly associated with cancer progression, including CRC, where it contributes to therapy resistance. Specifically, active STAT3 signaling has been shown to mediate resistance to chemoradiotherapy in the CRC cell lines. STAT3, along with both JAK1 and JAK2, play a role in the proliferation, viability, invasion, and migration of CRC cells by controlling the expression of various genes, including Bcl-2, p16^ink4a^, p21^waf1/cip1^, p27^kip1^, E-cadherin, VEGF, and MMPs [143]. Notably, it has been observed that anthocyanins have the potential to inhibit the activation of STAT3 and disrupt the JAK/STAT signaling pathway in CRC [84].

As potential sources of anthocyanin, blueberry and malvidin were found to possess inhibitory effects on the JAK/STAT3 pathway, thereby impeding the proliferation of cancer cells, as confirmed in a study [144]. Pretreatment with anthocyanin at a dosage of 100 mg/kg resulted in a significant reduction in hepatic JAK2/STAT3/P53 signaling activation in vivo [145]. Research focusing on MAPK and STAT-3 signaling revealed that at concentrations from 80 to 100 µM, delphinidin (an anthocyanidin) can effectively hinder the phosphorylation (suppression) of these transcription factors in HCT116 cells. The suppression of STAT-3, p38, and ERK1/2 phosphorylation, combined with the modulation of pro-apoptotic protein expression, is supposed to trigger the anti-cancer properties of delphinidin in CRC [111].

### 3.4. Anthocyanin on p53 Signaling Pathway

Talking about the p53 signaling pathway is highly significant in CRC due to its control over a wide range of cellular responses like DNA repair, cell cycle arrest, cellular senescence, and programmed cell death. In CRC, the TP53 gene is frequently mutated, with approximately 43–50% of tumors harboring missense mutations that not only impair the tumor-suppressing functions of wild-type p53 but also confer gain-of-function activities that promote cancer progression, stemness, and metastasis [146,147]. Additionally, epigenetic modifications, for example, DNA methylation and histone modifications, further compromise p53 function in CRC, even in cases where TP53 is not mutated, by inactivating downstream genes essential for p53 signaling [148]. One of many KLF4 can suppress p53 expression by directly acting on its promoter. The discovery was made that KLF4 functions to inhibit the apoptotic pathway dependent on p53 through the direct inhibition of *TP53* and direct suppression of *BAX* expression [149]. The loss of *Klf4* expression, observed in a subset of CRC cases, suggests its role as an early event in tumor development [150]. KLF4, a Zn finger transcription factor, functions as a tumor suppressor in CRC by upregulating p21WAF1/Cip1 and downregulating cyclin D1, thereby inhibiting cell proliferation and tumorigenesis [151]. Anthocyanin exhibited potent antiproliferative activities by promoting caspase-mediated cell death in a p53-independent manner, particularly in CRC stem cells, through the elevation of proteins mediating mitochondrial apoptosis, such as Bax and cytochrome C [152]. Also, the role of anthocyanins in modulating the KLF4 signaling pathway in CRC is less direct, but can be inferred and proven by several studies [153].

An investigation utilized anthocyanin-enriched purple-fleshed potatoes to elucidate its impact on colon cancer through both in vivo and in vitro studies involving colon cancer stem cells [138]. The anthocyanin extract derived from purple potatoes, when administered at a concentration of 5.0 μg/mL, demonstrated a significant reduction in proliferation rates by 63% and 32% in colon cancer stem cells possessing functional p53 and those with attenuated p53 via shRNA, respectively, as compared to the control group. These findings strongly indicate that the anthocyanin extract derived from purple potatoes effectively hinders the proliferation of colon cancer stem cells irrespective of their p53 status [138]. The same treatment led to an elevation in Cytochrome c levels regardless of the p53 status, suggesting that the activation of apoptosis may occur through the mitochondria-mediated apoptotic pathway [138]. Aronia berries are well known for their noteworthy levels of anthocyanin and proanthocyanidin [154,155]. Exposure to 50 µg monomeric anthocyanin/mL of Aronia extract for 24 h resulted in a 60% inhibition of growth in HT-29 [156]. Moreover, another examination illustrated that Aronia berry extract diminished the viability of CRC cells (SW480, HCT116). The Aronia berry extract impeded not only the proliferation, migration, survival, and invasion of CRC cells but also induced apoptosis in these cells. After the extract, an analysis of the genome-wide transcriptome identified 439 differentially expressed genes in both CRC cells. These genes were subsequently employed for pathway analysis of the p53 signaling pathway [157]. On top of that, investigation on DU-145 tumor xenografts in athymic nude mice revealed that daily oral consumption of anthocyanin (8 mg/kg) for 14 weeks resulted in a notable dose-dependent increase in apoptosis, significant reduction in p53 and Bcl-2 expressions (along with escalated Bax expression), and marked decrease in PSA and AR expressions. These results suggest that in the xenograft model, anthocyanin treatment considerably hinders tumor growth [115]. Moreover, not on CRC, but treatment with Cyanidin-3-O-glucoside (20 μM) for 24 h demonstrated inhibitory effects on the epithelial–mesenchymal transition process within this cellular context, leading to a notable reduction in the migratory and invasive capabilities of MDA-MB-231 and MDA-MB-468 cancer cells through the enhancement of *KLF4* expression at the protein level [121]. In general, the data indicate that anthocyanins can regulate the p53 signaling pathway and trigger apoptosis in CRC cells, underscoring their potential as adjunct therapeutic agents in CRC management.

### 3.5. Anthocyanin on mTOR Signaling Pathway

Research has shown that KLF4 could also influence the mTOR pathway and the p53-dependent cell-cycle pathway in CRC cell line HCT116. The overexpression of Klf4 was found to suppress mTOR pathway activity, leading to decreased levels of phosphorylated mTOR and p70S6K1 (pS371). Conversely, the downregulation of KLF4 resulted in a relative increase in phosphorylated mTOR and p70S6K1 (pS371) in CRC [158].

The treatment of cells with Cy3G at a concentration of 20 μM for 24 h exhibited inhibition of the epithelial–mesenchymal transition process. Furthermore, it significantly reduced the migratory and invasive capabilities of cancer cells by upregulating the expression of KLF4 at the protein level [121]. Additionally, investigations into the effects of anthocyanins on cancer cell survival and the AMPK/mTOR pathway revealed that anthocyanins extracted from Meoru exerted growth inhibitory effects by regulating the mTOR or GSK3β/β-catenin pathway in HT-29 colon and Hep3B cells, respectively [159]. Moreover, a separate study illustrated that anthocyanin from *Lycium ruthenicum* Murray inhibited cancer cell proliferation, suppressed migration and invasion, induced apoptosis, and caused G2/M phase cell cycle arrest through the activation of the AMPK/mTOR autophagy pathway [160]. These findings provide evidence that anthocyanins have the potential to enhance cancer management by modulating AMPK/mTOR or AMPK/Wnt signaling pathways.

### 3.6. Anthocyanin on PI3K-AKT Signaling Pathway

The PI3K/Akt/mTOR pathway plays an important role not only in controlling the proliferation and apoptosis of cancer cells but also in promoting normal and tumor angiogenesis [161]. AKT phosphorylation is responsible for driving cancer formation and progression by modulating the transcription factors that belong to the FOXO family, which are essential for suppressing growth and proliferation [162]. Current research suggests that anthocyanins possess the ability to prevent CRC cell proliferation and enhance apoptosis by influencing various signaling pathways, including the PI3K/AKT pathway that interacts with FOXO3a. Particularly, anthocyanins have demonstrated the capability to decrease PI3K protein expression and impede AKT phosphorylation, resulting in the activation of FOXO3a, thereby facilitating apoptosis and impeding tumor growth [163].

A study conducted on Caco-2 human colon carcinoma revealed that the anthocyanin extract, obtained from bilberries and blackcurrant at various concentrations, significantly reduced the proliferation of Caco-2 cells. This extract also triggered apoptosis through the activation of caspase-3 cleavage and increased the expression of cyclin-dependent kinase inhibitor 1 (p21Waf/Cif1) in a manner that depended on the dosage. Moreover, the anthocyanin extract exhibited a dose-dependent elevation in intracellular reactive oxygen species within Caco-2 cells, accompanied by a slight rise in the overall antioxidant status of the cells. These results further confirm the role of anthocyanins in enhancing apoptotic pathways mediated by FOXO [164]. Another study has also confirmed that anthocyanins can downregulate the PI3K/AKT pathway, which is recognized for its negative regulation of FOXO3a, thereby encouraging apoptosis and hindering tumor growth in human colon cancer HT29 cells [163]. Overall, the modulation of different co-factors in the PI3K/AKT signaling pathway by anthocyanins represents a promising strategy for CRC treatment, leveraging their natural bioactive properties to inhibit cancer cell growth and promote apoptosis through multiple molecular mechanisms.

### 3.7. Death Receptor Pathway

The death receptor pathway is a process that involves cell surface receptors that send apoptotic signals. Apoptosis, a well-orchestrated cellular demise mechanism crucial for preventing cancer progression, can be circumvented by malignant cells. AMP-activated protein kinase (AMPK) plays a pivotal role in maintaining cellular energy balance. The activation of AMPK can modulate various effector molecules involved in the control and progression of malignancies. AMPK activation can hinder the metabolic expansion of tumors by regulating energy levels, enforcing metabolic checkpoints, and restraining cell proliferation.

**Figure 4:**
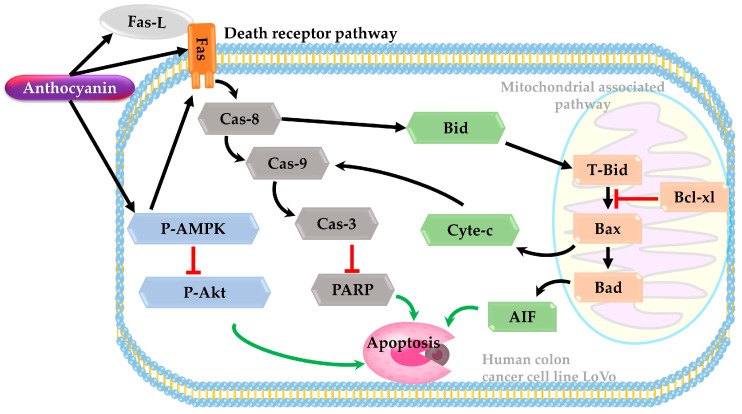
Effect of anthocyanin on initiating apoptosis in human colon cancer line. Black arrows indicate activation or promotion of processes. Green arrows represent the promotion of apoptosis, indicating pathways leading to cell death. Red blunt-ended lines indicate inhibition or blocking. Adopted form [165].

A study was carried out on the human colon cancer cell line LoVo using anthocyanin extract from Hibiscus flowers. The cells were treated with different concentrations of anthocyanin (1, 2, and 3 mg/mL) extracted from Hibiscus flowers for 24 and 48 h. The findings demonstrated a negative association between anthocyanin concentration, cell viability, and the generation of pro-apoptotic bodies. Treatment with anthocyanin extract led to mitochondrial damage and disintegration in LoVo cells in a dose-dependent manner, indicating a potential induction of apoptotic cell death in CRC cells through the intrinsic apoptotic pathway. Furthermore, higher concentrations of anthocyanin resulted in increased expression of p-AMPK and decreased expression of p-Akt, suggesting that anthocyanin extracts may modulate proteins associated with apoptotic pathways to trigger apoptosis (Figure 4) [165].

### 3.8. Anthocyanin on TGF-β/BMPs Signaling Pathway

Bone morphogenetic proteins (BMPs), which belong to the transforming growth factor-β (TGF-β) superfamily, serve as versatile cytokines that govern a wide array of biological processes. Recent research indicated that mutations in BMP receptor 1a and Smad4 in colon cancer highlighted that disruptions in BMP signaling significantly contribute to the development of intestinal cancer [134]. Mutations affect the TGF-β receptor type 2 (TGFBR2) due to a deficiency in mismatch repair, which is responsible for the development of CRCs with microsatellite instability, although this condition is linked to relatively improved survival rates [166]. Conversely, RUNX3 serves as a suppressor of gastric and colon tumors, operating in the pathway downstream of TGF-β. The tumor-inhibiting properties of RUNX3 are attributed to its capacity to dampen the transactivation of β-catenin/T-cell factors in the context of intestinal tumorigenesis [134]. The TGF-β/BMPs signaling pathway is significant in CRC where anthocyanin treatment could show a negative correlation with CRC. But there has been limited research conducted on the relationship between anthocyanin and the TGF-β/BMPs signaling pathway, as well as its impact on RUNX3.

Overall, anthocyanin has demonstrated significant potential in modulating several important signaling pathways in CRC, thereby influencing cellular mechanisms such as proliferation, apoptosis, and inflammation.

## 4. Potential of Bioengineering to Enrich Anthocyanin in Tomatoes

Tomatoes are considered a superior nutritional source due to their rich content of essential vitamins, minerals, and antioxidants, which contribute to various health benefits. Tomato is particularly high in lycopene, a potent antioxidant linked to decreased risks of several diseases as well as cancer [167]. The nutritional composition of tomatoes includes significant amounts of vitamin C, K, folate, and vitamin K, with around 95% water content, and the remaining 5% consisting of carbohydrates and fiber [167]. Moreover, its edible by-products, such as peels and seeds, are also nutritionally dense, containing 15.43% carbohydrates, 11.71% protein, and 5.4% lipids in peels, and 58.75% carbohydrates, 15.4% protein, and 22.2% lipids in seeds, with calorie values of 280.47 kcal/100 g and 472.8 kcal/100 g DM, respectively [168]. These by-products are also rich in minerals like K, Mg, Na, Fe, Zn, and higher levels of phenolic compounds, that contribute to its antioxidant properties [168].

Bioengineering tomatoes aiming to enrich anthocyanin content is driven by several compelling reasons, for instance, the health benefits, agricultural viability, and nutritional enhancement [18]. Traditional tomatoes are deficient in anthocyanins, but some wild relatives and specific genetic variants have been identified to accumulate these compounds in their sub-epidermal tissues [169,170]. By leveraging genetic engineering and crossbreeding techniques, scientists have successfully developed anthocyanin-rich tomato cultivars (Table 3). Research on introducing anthocyanin-enriching genes into tomatoes is showcased in the next paragraphs.

### 4.1. Introduce Delila (Del) and Rosea1 (Ros1) Genes in Tomato

**Figure 5:**
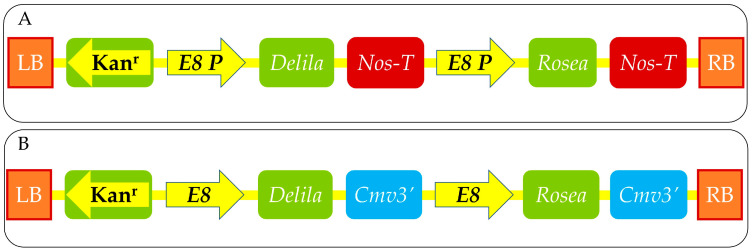
Map of T-DNA region of the binary vectors used for transformation. Schematic diagram expressing both *Delila (Del)* and *Rosea1 (Ros1)* under the control of the tomato fruit-specific E8 promoter in pGree II (**A**) [173,177] and pDEL.ROS (**B**) [18]. LB, left T-DNA border region; RB, right T-DNA border region; Kan^r^, *nptII* gene conferring kanamycin resistance under the control of the *nopaline synthase (nos)* promoter; Nos-T, *nos* terminator region; and *Cmv3*′, cauliflower mosaic virus terminator region.

*Del* and *Ros1* play crucial roles as regulatory genes in the process of anthocyanin biosynthesis, which is responsible for the vibrant red, purple, and blue colors observed in plants. These particular genes are also responsible for encoding transcription factors that serve to trigger the activation of a range of structural genes within the anthocyanin biosynthetic pathway. *Del* functions as a transcription factor of the basic helix–loop–helix (bHLH) type, whereas *Ros1* operates as a transcription factor of the R2R3-MYB type. Research has demonstrated that the simultaneous expression of both *Del* and Ros1 leads to a notable increase in anthocyanin accumulation across various plant species, such as snapdragon, tomato, petunia, and tobacco [178,179,180]. Two adopted schematic diagrams of cloning the anthocyanin genes for transformation into tomatoes are shown in Figure 5.

A study was conducted to add anthocyanin to tomato fruit through the activation of specific transcription factors. The *Del* and *Ros1* genes from snapdragon were introduced into the fruit of var. MicroTom [18]. The transgenic fruit exhibited normal development and manifested purple pigmentation towards the end of the mature green stage. Upon reaching maturity, various transgenic tomato lines displayed different phenotypes, ranging from medium (Del/Ros1Z) to high (Del/Ros1C and Del/Ros1Y) and very high anthocyanin accumulation (Del/Ros1N), with the highest concentrations averaging 2.83 ± 0.46 mg/g FW. In contrast, wild-type fruit showed minimal levels of anthocyanins. Purple fruit exhibited high anthocyanin levels in both the peel and flesh. The upregulation of the *Del* and *Ros1* genes’ expression increased the transcript levels of the majority of genes implicated in the biosynthesis of anthocyanin, along with the genes associated with side-chain alteration, such as a hypothetical anthocyanin acyltransferase, and two genes that could potentially play a role in the transport of anthocyanin into the vacuole, including a putative anthocyanin transporter [18].

**Figure 6:**
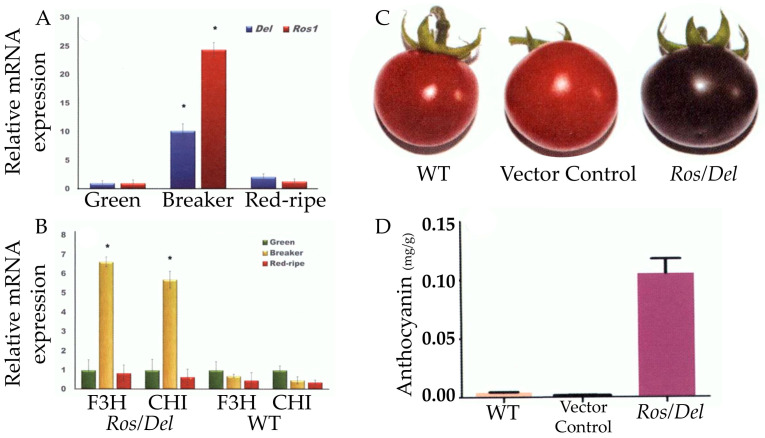
Del/Ros1l transgenic tomato. mRNA expression of *Del* and *Ros1* at different stages of transgenic tomato (**A**). mRNA expression of *F3H* and *CHI* in transgenic and WT tomatoes at different stages (**B**). WT, vector control, and transgenic fruits at mature stage (**C**). Anthocyanin content of WT, vector control, and transgenic tomato (**D**). The statistical significance was based on Student’s *t*-test, * *p* < 0.05. Adopted from [173].

Another group of researchers introduced *Ros1* and *Del* through *Agrobacterium*-mediated transformation into cv. Arka Vikas. This initiative helped to accumulate anthocyanin in transgenic lines of up to 0.1 mg/g (FW), which was 70–100 times higher than non-transgenic tomatoes [173]. In addition to anthocyanin, antioxidant capacity and carotenoid content were increased considerably in *Del* and *Ros1* transgenic tomatoes. They also revealed that the expression of the *CHI* and *F2H* genes became several-fold higher during the mature stage in transgenic fruits (Figure 6) [173]. CHI and F3H are key enzymes in flavonoid biosynthesis in plants [181,182].

To evaluate the organoleptic acceptance of transgenic tomatoes by end users, a study created CHI, Ros1, and Del transgenic *Solanum lycopersicum L.* tomato [174]. On average, the total flavanol content of the peel was elevated by 2.3-fold in the *Del/Ros* lines and 9.8-fold in the *CHI* lines compared to the wild-type tomatoes. The anthocyanin content of the peel ranged from 0.5 to 0.9 mg/g, whereas that of the flesh ranged from 0.03 to 0.08 mg/g in the transgenic line, which is significantly higher than wild-type and *CHI* tomatoes 165. In terms of sensory properties, the study did not find any difference between *Del/Ros1* and WT tomato in color, flavor, texture, and overall section [174]. It was indicated that Ros/Del transgenic tomato enhanced anthocyanin and flavanol without altering organoleptic properties.

The *PAP1* gene can enhance anthocyanin content in tomato shoots and also possibly produce anthocyanin production in fruits [183]. An individual study conducted on the *PAP1* gene alone with crossing the CHI transgenic line found that the skin of the transgenic tomato line that only introduced the *PAP1* gene showed a relatively higher abundance of anthocyanin, but was not significantly different than WT—12.3 µg/g and 8.17 µg/g, accordingly. In contrast, the generations of transgenic lines between the *CHI* × *PAP1* lines showed significantly higher total anthocyanin: 48.11 µg/g of tomato skin [183]. In addition to *PAP1*, *PAP2* expression also helped to produce anthocyanin. A study introduced *MYB90/PAP2* into tomato to enhance anthocyanin accumulation [171]. The expression of anthocyanin biosynthetic genes (*AtPAP2*) was increased. This resulted in the accumulation of anthocyanin in leaves (0.21 units/g FW) and flowers (0.3 units/g FW), but not in the fruits in the transgenic line. They concluded that *AtPAP2*’s only interaction with *SlJAF13* resulted in the prevention of anthocyanin accumulation in fruits [171]. This study suggested that the organ-specific expression of the anthocyanin accumulation gene and interaction with other genes also influence anthocyanin accumulation.

### 4.2. Introduce the SlMYB75 Gene in Tomato

MYB transcription factors play a role in different aspects of fruit quality by altering primary, secondary, and organic acid metabolism [184]. A research endeavor was designed to explore the potential function of SlMYB75 (also known as SlAN2) in enhancing tomato fruit quality, particularly in terms of anthocyanin accumulation. The overexpression of *SlAN2* in fruits resulted in a distinct orange color, rapid softening, and elevated levels of ethylene [185]. The researchers introduced *SlMYB75* into *Solanum lycopersicum* cv. Micro-Tom and conducted qRT-PCR analyses, which confirmed the presence of SlMYB75 transcripts in all tissues, although their expression in fruits was relatively low [175]. To address this issue, a sense construct of SlMYB75 was introduced into a tomato cultivar “Micro-Tom”, resulting in the development of three distinct transgenic homozygous lines. Certain generations of transgenic lines exhibited significantly elevated levels of *SlMYB75* expression in fruits, along with robust phenotypic characteristics. The overexpression of *SMYB75* found in transgenic homozygous lines resulted in a higher accumulation of anthocyanin (approximately 2.0 mg/g FW), whereas anthocyanin was absent in the wild-type tomato line [175].

*Del* and *Ros1* genes are key regulators in anthocyanin biosynthesis, enhancing the red, purple, and blue pigmentation in plants. Their simultaneous expression boosts anthocyanin accumulation significantly in various plants, including tomatoes, without affecting the organoleptic properties. Introducing *Del* and *Ros1* into tomatoes has resulted in increased anthocyanin, antioxidant capacity, and carotenoid content, with different phenotypic expressions across transgenic lines. Additionally, other genes like *PAP1* and *MYB* transcription factors also contribute to anthocyanin production, although their effects can vary depending on gene interactions and expression patterns. The overexpression of *SlMYB75* in tomatoes has shown a marked increase in anthocyanin content, demonstrating the potential for genetic enhancements to improve fruit quality.

## 5. Evidence of Anthocyanin-Enriched Tomato Extract as a Therapeutic Agent in CRC

**Figure 7:**
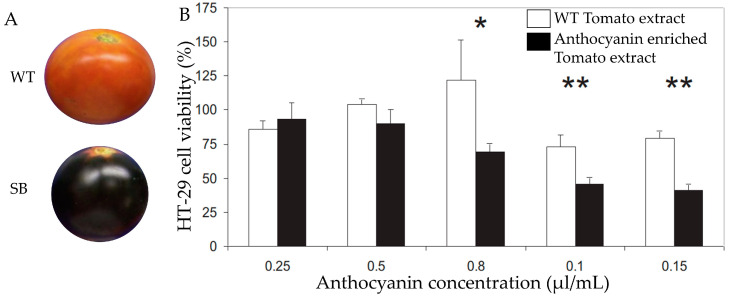
Wild-type and SB (Sun Black) tomato (**A**). Effect of tomato extract on CRC cell viability (**B**). Bars represent mean values SEM; * and ** indicate significant differences between genotypes within dose for *p* ≤ 0.05 and *p* ≤ 0.01, respectively. Adopted and modified from [186].

Numerous studies proved the role of anthocyanin extracted from different sources against CRC. To understand the importance of bioengineered anthocyanin-enriched tomatoes to fight against CRC, a few studies have been conducted [94]. For instance, a study has been conducted to investigate the consequence of extract from bioengineered anthocyanin-enriched tomato generated by breeding tomato varieties of anthocyanin fruit known as atroviolaceum (atv) against CRC. They treated colorectal adenocarcinoma cell HT-29 with five different anthocyanin concentrations (8.8, 17.5, 28.0, 35.0, and 52.5 mg/mL) of extracts from bioengineered tomato peel [186]. The extracts inhibited HT-29 cell proliferation in a dose-dependent manner. The maximum inhibition was observed after 24 h exposure to anthocyanin-enriched extract, which contains 28 μg/mL of anthocyanin. On the contrary, the survival rate of HT-29 cells exposed to an equivalent amount of wild-type tomato extract consistently hovered at 80–90% [186] (Figure 7). Through this finding, it has been demonstrated that bioengineered anthocyanin-enriched tomatoes can be a potential candidate fruit to supply anthocyanin and mitigate anthocyanin deficiency-related disease.

**Figure 8:**
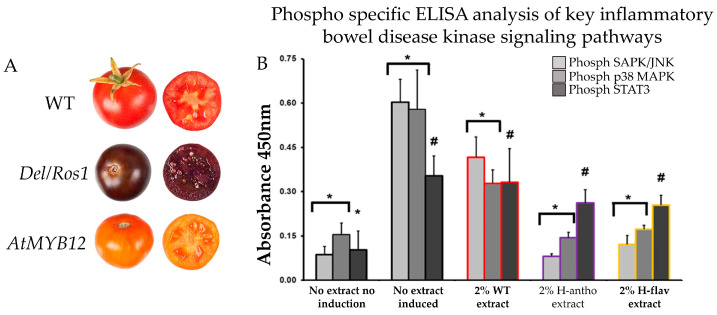
Bioengineered anthocyanin- and anthocyanin-enriched tomato (**A**). Effect of anthocyanin- and flavonoid-enriched tomato extract on C57BL/6 mice’s colonic epithelial cell (**B**). * Statistically significant (*p* < 0.05). # Not statistically significant (*p* > 0.05). Adopted and modified from [184,187].

A further investigation was carried out on the colonic epithelial cell (CEC) of male C57BL/6 mice. The CEC cells were subjected to treatment either with and/or without the addition of tomato extracts (2% *v*/*v*) derived from *Del/Ros1* (H-antho) and *AtMYB12* (H-flav) transgenic anthocyanin-enriched tomato lines [184]. Utilizing an ELISA assay with phospho-epitope specific capture antibodies, in conjunction with primary murine CEC lysates, the activation status of NF-κB, SAPK/JNK, p38 MAPK, and STAT3 kinases was assessed. The introduction of high anthocyanin tomato extract resulted in an 87% decrease in SAPK/JNK activity in comparison to controls with no tomato extract. Similarly, the high anthocyanin extracts led to a 75% reduction in p38 MAPK activation, when compared to the control group without tomato extract (Figure 8). Notably, the high anthocyanin extract exhibited a significant impact on p38 MAPK activation as well [187]. In summary, the outcomes of the study unequivocally showcased the influence of anthocyanin on the signaling pathway within colon cells.

## 6. Implications, Limitations, and Future Research

Bioengineering tomatoes that are enriched in high anthocyanins could provide a widely accessible and cost-effective dietary intervention to mitigate the global burden of CRC. This approach also aligns with the increasing demand for functional foods that offer therapeutic benefits. By integrating anthocyanin-enriched tomatoes into the diet, we can harness their preventive and therapeutic abilities, offering a promising strategy to reduce CRC incidence and improve patient outcomes worldwide.

However, there are several challenges associated with the bioengineering of tomatoes to produce higher levels of anthocyanins. The total anthocyanin concentration in existing bioengineered anthocyanin-enriched tomatoes may not be high enough. Therefore, it is necessary to identify and introduce new anthocyanin-synthesizing genes or establish a genome-editing technology of existing anthocyanin-synthesizing genes in tomatoes, which is challenging. Additionally, open-field environmental factors (soil pH, temperature, and nutrition availability) for large production could be a threat to maximizing anthocyanin accumulation in bioengineered tomatoes. This requires comprehensive studies to understand how different environmental factors influence anthocyanin biosynthesis and to develop best practices for cultivation. On top of that, bioengineered crops have already been accepted by several governments, but some are still in the process of approval, and a few countries did not approve them at all. Thus, acceptance by the general public remains a concern.

Future research on the effect of anthocyanin-enriched bioengineered tomatoes on CRC should focus on the key areas to maximize the therapeutic potential and health benefits of these bioengineered plants. Firstly, identifying and introducing new genes that significantly enhance anthocyanin accumulation in tomatoes is crucial. Advanced genetic engineering techniques, such as genome editing with CRISPR-Cas9, can be utilized to modify regulatory genes involved in anthocyanin biosynthesis, ensuring higher concentrations of these beneficial compounds. Secondly, comprehensive studies should be conducted to evaluate all potential health outcomes associated with the consumption of bioengineered anthocyanin-rich tomatoes. This includes examining antioxidant activity, anti-inflammatory effects, and potential impacts on gut microbiota. Thirdly, a deep investigation into the molecular pathways influenced by high anthocyanin levels in bioengineered tomato extracts is necessary, particularly focusing on their role in reducing inflammation and other mechanisms in CRC. This could involve the integration of omics (transcriptomic, proteomic, epigenomic, and metabolomic) techniques to uncover the interactions between anthocyanins and cellular pathways. Lastly, conducting studies on large animal models and eventually human clinical trials will be essential to establish the efficacy and safety of these bioengineered tomatoes, ensuring wider acceptance and potential integration into dietary recommendations for cancer prevention and therapy.

## 7. Conclusions

Given the critical need for affordable and effective solutions to combat CRC, it is essential to explore protective measures against inflammation. Among natural compounds, anthocyanins have demonstrated significant potential in fighting CRC. Tomatoes enriched with anthocyanins through bioengineering present a promising source of this beneficial compound due to their affordability, worldwide availability, and ease of cultivation.

Bioengineered anthocyanin-enriched tomatoes offer a viable option for delivering the health benefits of anthocyanins to a broad population, including those who may not have access to other anthocyanin-rich fruits. The widespread acceptance and use of these high anthocyanin bioengineered tomatoes could play a crucial role in the global fight against CRC.

## Figures and Tables

**Table 1:** Major sources of anthocyanin.

Scheme		Subsets of Anthocyanin		References
Fruits	TAC	Name of the Compounds	Concentration	
Açaí	732 mg/100 g	Cyanidin-3-O-glucoside	133.25 mg/100 g DW	[30,31]
		Cyanidin-3-O-rutinoside	225.61 mg/100 g DW	
Blackcurrant	294.38 mg/100 g	Delphinidin-3-O-glucoisde	8.58 mg/100 g FW	[32,33]
		Delphinidin-3-O-rutinoside	42.73 mg/100 g FW	
		Cyanidin-3-O-glucoside	2.99 mg/100 g FW	
		Cyanidin-3-O-rutinoside	30.11 mg/100 g FW	
Queen Garnet plum	277 mg/100 g	Cyanidin-3-O-glucoside	17.00 mg/100 g FW	[34,35]
Blueberry	275.86 mg/100 g	Cyanidin-3-O-glucoside	0.01 mg/100 g	[36,37]
Sweet potato	223 mg/100 g	Cyanidin 3-p-hydroxybenzoyl sophoroside-5-glucoside	85.80 mg/100 g	[38,39]
		Cyanidin 3-(6‴-caffeoyl sophoroside)-5-glucoside	33.90 mg/100 g	
		Peonidin 3-p-hydroxybenzoyl sophoroside-5-glucoside	710.00 mg/100 g	
		Peonidin 3-(6‴-caffeoyl sophoroside)-5-glucoside	229.00 mg/100 g	
		Cyanidin 3-feruloyl sophoroside-5-glucoside	204.00 mg/100 g	
		Peonidin 3-feruloyl sophoroside-5-glucoside	712.00 mg/100 g	
		Cyanidin 3-caffeoyl sophoroside-5-glucoside	1310.00 mg/100 g	
		Cyanidin 3-sophoroside-5-glucoside	444.00 mg/100 g	
		Cyanidin 3-dicaffeoyl sophoroside-5-glucoside	1220.00 mg/100 g	
		Cyanidin 3-caffeoyl-p-gydroxybenzoyl sophoroside-5-glucoside	1480.00 mg/100 g	
		Peonidin-3-caffeoyl sophoroside-5-glucoside	3250.00 mg/100 g	
		Cyanidin 3-caffeoyl-feruloyl sophoroside-5-glucoside	1620.00 mg/100 g	
		Peonidin 3-dicaffeoyl sophoroside-5-glucoside	5790.00 mg/100 g	
		Peonidin 3-caffeoyl-p-hydroxybenzoyl sophoroside-5-glucoside	7570.00 mg/100 g	
		Peonidin 3-caffeoyl-feruloyl sophoroside-5-glucoside	6920.00 mg/100 g	
		Peonidin 3-caffeoyl-p-coumaryl sophoroside-5-glucoside	559.00 mg/100 g	
		Peonidin 3-feruloyl-p-hydroxybenzoyl sophoroside-5-glucoside	581.00 mg/100 g	
		Peonidin 3-coumaryl-p-hydroxybenzoyl sophoroside-5-glucoside	181.00 mg/100 g	
		Peonidin 3-(6″, 6‴-diferuloyl sophoroside)-5-glucoside	243.00 mg/100 g	
Cherry	223 mg/100 g	Cyanidin 3-O-galactoside	22.62 mg/100 g DW	[40]
Raspberries	211.3 mg/100 g	Cyanidin-3-O-sophoroside	25.40 mg/100 g	[41,42]
Purple corn	194.47 mg/100 g	Cyanidin-3-O-glucoside	41.45 mg/100 g DW	[43,44]
Red cabbage	191.37 mg/100 g	Cyanidin-3-diglucoside-5-glucoside	58.00 mg/100 g DW	[45,46]
		Cyanidin-3-(sinapoyl)(sinapoyl)-diglucoside-5-glucoside	26.00 mg/100 g DW	
		Cyanidin-3-(feruloyl)(sinapoyl)-diglucoside-5-glucoside	18.00 mg/100 g DW	
		Cyanidin-3-(feruloyl)(feruloyl)-diglucoside-5-glucoside	17.00 mg/100 g DW	
		Cyanidin-3-(sinapoyl)-diglucoside-5-glucoside	18.00 mg/100 g DW	
		Cyanidin-3-(p-coumaroyl)-diglucoside-5-glucoside	19.00 mg/100 g DW	
Tomato	120 mg/100 g	Cyanidin-3-O-galactoside	0.03 mg/100 g FW	[47,48]
		Cyanidin-3-O-rutinoside	0.11 mg/100 g FW	
		Cyanidin-3-(6-caffeoyl)-glucoside	0.09 mg/100 g FW	
		Delphinidin-3-O-glucoside	2.00 mg/100 g FW	
		Delphinidin-3-rutinoside-5-glucoside	0.10 mg/100 g FW	
		Delphinidin-3,5-O-diglucoside	0.20 mg/100 g FW	
		Delphinidin-3-O-rutinoside	7.50 mg/100 g FW	
		Peonidin-3-O-rutinoside	0.07 mg/100 g FW	
		Peonidin-3-O-(6-O-p-counmaryl)-glucoside	0.07 mg/100 g FW	
		Petunidin-3-O-glucoside	0.19 mg/100 g FW	
		Petunidin-3-O-rutinoside	0.51 mg/100 g FW	
		Malvidin-3-)-glucoside	0.00 mg/100 g FW	
		Malvidin-3-O-rutinoside	0.09 mg/100 g FW	
		Cyanidin-3-(sinapoyl)-diglucoside-5-glucoside	12.00 mg/100 g DW	
		Cyanidin-3-(feruloyl)-diglucoside-5-glucoside	14.00 mg/100 g DW	
Blackberry	102.7 mg/100 g	Cyanidin-3-O-glucoside	40.43 mg/100 g	[49]
		Cyanidin-3-O-sophoroside	42.30 mg/100 g	
		Cyanidin-3-O-xyloside	0.11 mg/100 g	
		Pelargonidin-3-O-glucoside	0.79 mg/100 g	
		Petunidin-3-O-glucoside	0.01 mg/100 g	
		Cyanidin-3-O-rutinoside	18.63 mg/100 g	
		Peonidin-3-O-galactoside	0.07 mg/100 g	
		Peonidin-3-O-glucoside	0.36 mg/100 g	

**Table 2:** Effect of anthocyanin on modulation of transcription factors involved in CRC.

Pathways in CRC	Transcription Factor	Effect of Anthocyanin on the Transcription Factors	References
NFκB signaling pathway	NFκB	▪ Anthocyanin increased IκBα and interrupted NFκB alpha activity. ▪ Study model: BV2 cell. ▪ Dose: 100 µg/mL.	[107,108,109,110]
Wnt/β-catenin signaling pathway	NFκB		
JAK/STAT signaling pathway	STAT3	▪ Anthocyanin decreased STAT3 activity. ▪ Study model: HCT116. ▪ Dose: 80–120 µM.	[111,112]
p53 signaling pathway	p53	▪ Anthocyanin increased p53 expressions and apoptosis. ▪ Study model: DU-145 cells. ▪ Dose: 8 mg/kg body weight.	[113,114,115,116]
NF-κB signaling pathway	p53		
PI3K-AKT signaling pathway	FOXO3a	▪ Black seed rich in anthocyanin treatment decreased FOXO3 levels by 74% and induced FOXO3 phosphorylation. ▪ Study model: HepG2 Cell.	[117,118,119,120]
Wnt/β-catenin signaling pathway	FOXO3a		
mTOR signaling pathway	KLF4	▪ Anthocyanin (Cy3G) increased KLF4 protein level. ▪ Study model: MDA-MB-231 and MDA-MB-468 cells. ▪ Dose: 20 μM.	[121,122,123]
Wnt/β-catenin signaling pathway	KLF4		
p53 signaling pathway	KLF4		
TGF-β/BMPs signaling pathway	RUNX3	-	[124]
Wnt/β-catenin signaling pathway	RUNX3	-	

**Table 3:** Bioengineered tomato and anthocyanin accumulation.

Transgenic Lines	Maximum Anthocyanin Contents	Other Function Enhanced	References
*Delila* and *Rosea1* transgenic *Solanum lycopersicum* cv. MicroTom	Fruits 2.83 ± 0.46 mg/g FW	▪ Increased the activity of the water-soluble fraction.	[18]
*AftAft/atvatv* purple line (SB)	Whole Mature fruit 1.2 mg/g DW and 7.1 mg/100 g FW.	▪ Increased chlorogenic, rutin, carotenoid, phenolic, vitamin C, and antioxidant activities.	[47]
*MYB90/PAP2* transgenic tomato cv. Micro-Tom	Leaves 0.21. units/g FW. Flower > 0.3 unit/g FW.	▪ Reduced fruit size. ▪ Decreased length and number of roots.	[171]
*CHI* × *PAP1* transgenic *Solanum lycopersicum* L. cv. Rubion	Fruits skin: 48.11 µg/g	▪ Increased flavanol.	[172]
*Del* and *Ros1* tomato cv. Arka Vikas	Fruits 0.01 mg/g FW	▪ Other biochemicals remained unchanged.	[173]
CHI, Delila and Rosea1 transgenic *Solanum lycopersicum* L.	Peel 0.5–0.9 mg/g Flesh 0.03–0.08 mg/g	▪ Increased flavanol.	[174]
SlMYB75-OE *Solanum lycopersicum* cv. Micro-Tom	Fruits 2.0 mg/g FW	▪ Delayed ripening. ▪ Better Stress responses, secondary metabolism, and phytohormone signaling pathways. ▪ Increased volatile compounds.	[175]
Del/Ros1 transgenic purple tomatoes	Fruit 5.2 g/kg DW, Peel 5.1 g/kg DW, and Flesh 5.8 g/kg DW.	▪ Not observed.	[176]
Del/Ros1 bred Moneymaker tomato	Fruits 0.7–0.8 g/kg DW (green) and Fruits 1.3–3.0 g/kg DW (mature)	▪ Increased antibacterial activity.	[177]

## Data Availability

The original contributions presented in the study are included in the article, further inquiries can be directed to the corresponding author.
